# Syphilis

**Published:** 1872-12

**Authors:** 


### Syphilis.

Dr. Ricord, in a recent conversatione (the Doctor) was quite emphatic in the declaration that syphilis could be "perfectly, radically cured." The following, in reply to a question by Dr. Gross, is worthy of careful consideration:

Dr. Ricord said his opinion was that a soft chancre, when accurately diagnosed, never gave rise to constitutional disease. This was a law as absolute as possible. But they must be careful, or errors of diagnosis might be made. It was not always easy to establish the difference between soft and hard chancre, but when the diagnosis was certain, they might be sure they would not have any constitutional disease after the soft chancre. On the contrary, even as long as six months after hard chancre secondary symptoms would appear. This was one of the most clearly established facts in practice. But the hardness of the chancre was not always well marked *(bien formulee)*; it might be very superficial in those varieties that were attended with excoriation. When there was a something like parchment at the base, a chancre was very easily taken to be soft, but was not so: and he had had cases sent to him as instances of soft chancre which had been followed by secondary symptoms, but which were well characterized by the parchment-like base. However, there was a symptom of more value than the parchment base, a symptom that was one of the most important witnesses to constitutional affection, and that was the non-inflammation of the glands—they were cold and dull. In general several of them became enlarged; it was very seldom that only one was found to swell after hardened chancre; and not only were the glands swollen, but the enlarement frequently occurred on both sides, in both groins. The enlargement of the glands was of much value as a character of hardened chancre. The enlarged glands appeared very early, even during the first fortnight of the existence of the sore. With the soft chancre the glands did not always swell; in a great many cases there was no swelling. They would never find a real hard chancre without swelling of the glands; and they would often find many cases of soft chancre with swelling, these cases depending upon surgeons confounding the hard chancre with thickening dependent upon inflammatory infiltration of the tissue immediately around the sore. But if the glands should swell after soft chancre, it was probably that suppuration would come on. With hard chancre there was no inflammation and no suppuration. The older writers directed their efforts to cause an indurated sore to suppurate, in the belief arising from the practical observation that when a bubo suppurated there was no constitutional disease, and therefore they were under the belief that the poison was thrown out of the body. In their quaint way of putting the fact, "they did not like to shut up the wolf within the fold." But they could not bring on specific suppuration in the case of indurated glands; it was impossible. He had tried all means of doing it, and could not succeed in the cases of specific suppuration. In the instance of soft chancre what had they to do—await the occurrence of suppuration, which might either be attended by simply inflammatory or specific bubo. When a patient consulted him (M. Ricord) suffering from soft chancre he said to him, "Be quiet; you may have a bubo; that will suppurate, but your constitution will be unaffected; you will not be liable to secondary symptoms." With a hard chancre he could predict indurated glands, attended by constitutional symptoms, within six months, provided proper treatment were not followed. He would add, that when it was decided that the case was one of hard chancre or soft chancre, the treatment was very simple. When there was a doubt as to the nature of the chancre, he waited till some characteristic symptom arose. But there was cases in which the existence of a soft chancre did not prevent a patient from contracting a hard chancre. The patient might have the two species at the same time, contracted from the different sources. The two species, hard and soft chancres, do not depend upon the difference in the ground, but a difference in the seed *(contagium)*. So that the new comer who had relations with a woman suffering from the two species could take his choice. If the patient had a true undurated chancre and well diagnosed secondary symptoms, he might catch the soft chancre as often as he pleased, and it would be unattended with specific constitutional disturbance.

